# A Mitochondria-Associated Oxidative Stress Perspective on Huntington’s Disease

**DOI:** 10.3389/fnmol.2018.00329

**Published:** 2018-09-19

**Authors:** Ju Zheng, Joris Winderickx, Vanessa Franssens, Beidong Liu

**Affiliations:** ^1^Department of Biology, Southern University of Science and Technology, Shenzhen, China; ^2^Department of Biology, Functional Biology, KU Leuven, Heverlee, Belgium; ^3^Department of Chemistry and Molecular Biology, University of Gothenburg, Gothenburg, Sweden; ^4^State Key Laboratory of Subtropical Silviculture, School of Forestry and Biotechnology, Zhejiang A&F University, Hangzhou, China; ^5^Center for Large-scale Cell-based Screening, Faculty of Science, University of Gothenburg, Gothenburg, Sweden

**Keywords:** Huntington’s disease, Huntingtin, neurodegeneration, yeast, oxidative stress

## Abstract

Huntington’s disease (HD) is genetically caused by mutation of the Huntingtin (*HTT*) gene. At present, the mechanisms underlying the defect of *HTT* and the development of HD remain largely unclear. However, increasing evidence shows the presence of enhanced oxidative stress in HD patients. In this review article, we focus on the role of oxidative stress in the pathogenesis of HD and discuss mediators and potential mechanisms involved in mutant HTT-mediated oxidative stress generation and progression. Furthermore, we emphasize the role of the unicellular organism *Saccharomyces cerevisiae* in investigating mutant HTT-induced oxidative stress. Overall, this review article provides an overview of the latest findings regarding oxidative stress in HD and potential therapeutic targets for HD.

## Introduction

### Huntington’s Disease

Huntington’s disease (HD) is a neurodegenerative disorder inherited in an autosomal dominant pattern. The symptoms in affected individuals include emotional problems, psychiatric disturbances and a decline in the ability to control movements and thinking.

Mutation of the Huntingtin (*HTT*) gene causes HD. An expansion of CAG repeats was found in the DNA sequence of mutant *HTT*, resulting in a polyQ expansion in the encoded mutant HTT protein (MacDonald et al., 1993). This polyQ expansion of HTT has been proven to trigger misfolding/aggregation of HTT and cytotoxicity to cells (DiFiglia et al., 1997; Penney et al., 1997; Krobitsch and Lindquist, 2000). The CAG-segment in the *HTT* gene is repeated approximately 25 times in healthy individuals. In contrast, individuals with 40–50 CAG repeats in the *HTT* gene usually develop adult-onset HD and the presence of more than 60 CAG repeats of *HTT* tends to result in the development of juvenile HD (MacDonald et al., 1993; Langbehn et al., 2004). Despite the well-studied genetic cause of HD, the mechanisms by which polyQ expansion induces cytotoxicity and neuronal death are not yet fully understood. Expression of the N-terminal fragment of HTT with a polyQ expansion results in polyQ protein aggregation and produces neurological symptoms (Lunkes et al., 2002). Therefore, both full-length and truncated HTT containing a polyQ expansion exceeding 36Q are defined as mutant HTT (mHTT) in this review article. As the proline-rich region is critical for polyQ expansion-induced toxicity in a yeast model (Dehay and Bertolotti, 2006; Duennwald et al., 2006), we will emphasize the presence/absence of the proline-rich region within a mHTT fragment in the yeast model.

In addition to the effect of polyQ-expanded HTT, CAG repeats within HTT mRNA were also identified as a toxic species. Importantly, the toxic effect of CAG repeat within mRNA are also length dependent. CAG repeats in mRNA may serve as a template for formation of RNA foci (Jain and Vale, 2017). Furthermore, expanded CAG repeats sequester functional proteins and cause gene expression perturbations (Martí, 2016). Moreover, non-polyQ-expanded homomeric proteins were reported to accumulate in the HD human brain and were demonstrated to be toxic to neural cells in a consistent, length-dependent manner (Bañez-Coronel et al., 2015).

### Oxidative Stress

Cells possess complicated mechanisms to ensure their proper functioning. These mechanisms are responsible for the elimination of harmful by-products generated during biological processes. However, when these cellular systems fail to maintain the balance between generations of toxic by-products and scavenging, stress occurs, and this may result in cellular cytotoxicity. Oxidative stress is the imbalance between reactive oxygen species (ROS)/reactive nitrogen species (RNS) generation and the biological antioxidant defense system. ROS/RNS are very active species that interact with a number of cellular macromolecules, resulting in a reversible alteration of their molecular structure and function that causes a subsequent cellular response. Accumulation of ROS/RNS in cells leads to damage of proteins, DNA and lipids and further damages tissues and organs, and these changes contribute to the pathogenesis of many diseases (Sies, 1991; Yu, 1994; Weidinger and Kozlov, 2015).

Endogenous ROS mainly originates from mitochondria during the synthesis of ATP and the transfer of electrons, derived from the mitochondrial respiratory reactions, to the electron transport chain. Part of the electrons generated during this process interact with O_2_, thereby generating superoxide. It was estimated that approximately 2% of the total amount of O_2_ that is consumed by mitochondria is involved in ROS generation (Chance et al., 1979; Sabharwal and Schumacker, 2014). In addition, ROS is also produced by NADPH oxidase (NOX) within the cytoplasm (Valencia et al., 2012).

In this review article, we address the ROS production upon expression of mutant HTT and how this increased ROS level contributes to cytotoxicity and neuronal death.

## Mitochondrial Dysfunction Contributes to HD Pathogenesis

**Figure 1:**
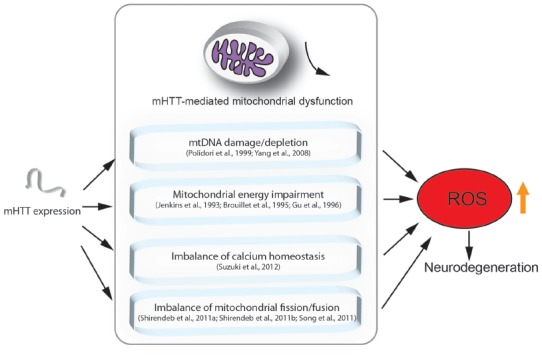
Mutant HTT (mHTT) induced mitochondria-mediated reactive oxygen species (ROS) accumulation. This figure summarizes the mitochondria-associated dysfunction caused by expression of mHTT. mHTT directly contacts the mitochondrial membrane and disturbs calcium homeostasis (Suzuki et al., 2012). mHTT causes mitochondrial dysfunction by damaging mitochondrial DNA (mtDNA; Polidori et al., 1999; Yang et al., 2008). mHTT interrupts mitochondrial fission/fusion and interferes with mitochondrial energy metabolism (Jenkins et al., 1993; Brouillet et al., 1995; Gu et al., 1996; Shirendeb et al., 2011a; Song et al., 2011). Mitochondrial defects result in ROS accumulation, which further leads to apoptosis and cell death.

Although the mechanisms underlying HD are still unclear, there is evidence showing that mitochondrial dysfunction indeed plays a crucial role in the pathogenesis of HD (Lin and Beal, 2006; Quintanilla and Johnson, 2009; Damiano et al., 2010; Costa and Scorrano, 2012; Guedes-Dias et al., 2016; Carmo et al., 2018). Here, we highlight the major findings regarding the role of mitochondrial loss or dysfunction in HD pathogenesis (Figure 1).

### Evidence for Mitochondrial Dysfunction in HD Pathology

Oxidative stress causes damage to mitochondrial DNA (mtDNA) during ageing. In addition, researchers have provided evidence that mtDNA damage is implicated in the pathogenesis of HD (Polidori et al., 1999; Yang et al., 2008). In this respect, an increase in both the basal levels of mitochondria-generated ROS and mtDNA lesions and a simultaneous decrease in spare respiratory capacity were observed in striatal cells expressing mHTT. As mtDNA is a major target of the oxidative stress associated with mHTT, researchers found that the abundance of mtDNA decreases dramatically in mHTT cells compared to wild-type cells (Siddiqui et al., 2012). In addition, mtDNA depletion in leukocytes was reported in patients with polyQ diseases, and the level of mtDNA content was negatively correlated to the number of polyQ repeats in the mHTT (Liu et al., 2008). A mitochondrial biology study showed that a selective mtDNA depletion coincides with an increased vulnerability of the striatum in transgenic HD mice (Hering et al., 2015). In addition, the copy number of mtDNA is significantly lower in peripheral HD leukocytes and transgenic HD R6/2 mice (Petersen et al., 2014). Moreover, expression of mHTT impairs the mitochondrial disulfide relay system (MDRS), which is accompanied by a decreased copy number of mtDNA and the accumulation of mtDNA deletions (Napoli et al., 2013).

**Figure 2:**
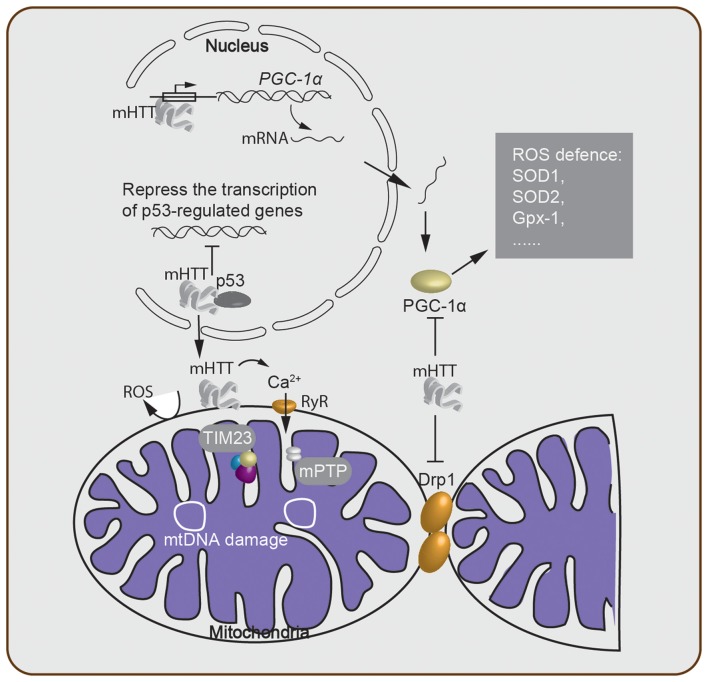
Oxidative stress-associated cellular processes involved in Huntington’s disease (HD) pathogenesis. mHTT acts within the nucleus and either suppresses transcription of critical genes and downregulates mitochondrial function or directly alters the expression level of mitochondrial biogenesis-associated proteins such as PGC-1α. This alteration of the PGC-1α protein level then further regulates the ROS defense system in the cytosol. The mHTT-mitochondria interaction generates ROS through many processes, including Ca^2+^ leakage through the ryanodine receptor (RyR). Failure of Ca^2+^ homeostasis triggers the opening of the mitochondrial permeability transition pore (mPTP), which disturbs the ATP level and enables release of cytochrome* c*. Meanwhile, mitochondrial protein import is impaired due to the binding of mHTT to the TIM23 complex. mtDNA damage and depletion within the mitochondria elevate ROS production. Lastly, cytosolic mHTT inhibits mitochondrial fission–fusion by interacting with Drp1.

Mitochondria are key organelles that participate in the regulation of intracellular Ca^2+^ homeostasis (Giacomello et al., 2007). When the Ca^2+^ level in the cytosol is high, the extra Ca^2+^ is sequestered into inactive calcium-phosphate complexes. On the other hand, when the Ca^2+^ level is low, Ca^2+^ is released back into the cytosol (Nicholls and Budd, 2000; Nicholls, 2005). Researchers found that calcium transport between the mitochondria and cytoplasm occurs through the ryanodine receptor (RyR), an endoplasmic reticulum-resident Ca^2+^ channel. Inhibitors of the RyR attenuated cell death induced by mHTT, while co-expression of the RyR enhanced HTT toxicity (Figure 2). Furthermore, Ca^2+^ leakage was observed in striatal and cortical neurons from R6/2 HD model mice (Suzuki et al., 2012). These observations show that maintaining an appropriate Ca^2+^ concentration within the cytoplasm is vital for cell viability. However, failure of the Ca^2+^ homeostasis buffering system or an overload of Ca^2+^ can lead to the opening of a non-specific pore in the inner mitochondrial membrane, known as the mitochondrial permeability transition pore (mPTP). The opening of the mPTP can disturb the ATP level within mitochondria. Since a proper ATP level is critical for cells to function properly, opening the mPTP potentially kills the cell (Krieger and Duchen, 2002; Halestrap, 2006; Rasola et al., 2010).

Increasing evidence shows that a defect in mitochondrial Ca^2+^ handling contributes to HD pathogenesis (Brustovetsky, 2016). Lower membrane potential and higher sensitivity to calcium loads were found in lymphoblast mitochondria from HD patients and brain mitochondria from transgenic mice expressing mHTT (Panov et al., 2002). Another study showed that mHTT interacts with the outer mitochondrial membrane and directly induces opening of the mPTP, which is accompanied by a significant release of cytochrome *c* (Choo et al., 2004). This release of cytochrome *c* has been shown to initiate apoptosis (Green and Reed, 1998). Addition of cyclosporin A, an inhibitor of the mPTP, prevented the release of cytochrome *c* (Figure 2). Moreover, a defect in calcium handling not only resulted in the opening of the mPTP but also caused higher levels of mtDNA damage in HD cells. The same effect was observed with elevated superoxide production, suggesting a new link between an imbalance in mitochondrial Ca^2+^ signaling and elevated oxidative DNA damage in HD (Wang et al., 2013). In addition, it was also shown that the first 17 amino acids and the polyQ repeat region of HTT, cause mitochondrial dysfunction in a calcium-homeostasis disrupted manner (Rockabrand et al., 2007). Furthermore, loss of mitochondrial integrity was observed during ageing and disease progression (Hughes and Gottschling, 2012; Szklarczyk et al., 2014), and the integrity of the mitochondrial cristae were found to be disrupted in HD, which was potentially caused by the oligomerization of OPA1, an optic atrophy protein (Hering et al., 2017). Overall, these results indicate that the imbalance in calcium homeostasis and mitochondrial dysfunction contribute to HD pathogenesis.

### Mediators of mHTT-Mediated Mitochondrial Dysfunction

It has been hypothesized that the cytotoxicity mediated by polyQ expansion of mHTT is caused by altering the expression level of cellular factors (McCampbell et al., 2000; Schaffar et al., 2004). The tumor suppressor protein, p53, was found to interact with mHTT, and the mHTT-p53 interaction represses the transcription of genes regulated by p53 (Steffan et al., 2000). Furthermore, activation of p53 increased both mHTT mRNA and protein expression, thereby suggesting that p53 is a modulator of the processes involved in HD development (Feng et al., 2006). On the other hand, suppression of p53 reversed the mHTT-induced mitochondrial depolarization and cytotoxicity in HD cells (Bae et al., 2005), indicating that p53 plays an important role in mediating mitochondrial dysfunction caused by mHTT. Moreover, an interaction between mHTT and the mitochondrial protein import complex TIM23 was reported. This mHTT-TIM23 interaction causes a mitochondrial protein import defect and induces neuronal death (Yano et al., 2014).

Other studies also demonstrated that ROS production in HD is mediated by PGC-1α, a transcriptional co-regulator of mitochondrial biogenesis, metabolism, and antioxidant defenses (Johri et al., 2013). mHTT caused an increase in PGC-1α-mediated oxidative stress via multiple mechanisms (Figure 2): (1) mHTT binds to the promoter sequences of PGC-1α and reduces the transcription level of PGC-1α (Chaturvedi et al., 2012). (2) mHTT binds directly to PGC-1α, suppresses the activity of PGC-1α, which in turn, reduces the expression level of downstream targets of PGC-1α, including mitochondrial uncoupling proteins and antioxidants such as copper/zinc superoxide dismutase (SOD1), manganese SOD (SOD2), and glutathione peroxidase (Gpx-1; St-Pierre et al., 2006). SODs are major antioxidant enzymes that scavenge O_2-_ radicals in the antioxidant system (Gandhi and Abramov, 2012; Kim et al., 2015). Thus, the lower expression level of SOD1, the more SOD2 and Gpx-1 contribute to the mitochondrial dysfunction, which enhances the pathology of HD. (3) mHTT interacts with Drp1 (Shirendeb et al., 2011b), thereby interfering with the mitochondrial dynamics by disrupting the balance of the mitochondrial fission–fusion processes (Shirendeb et al., 2011a; Song et al., 2011). Indeed, overexpression of PGC-1α rescued HD neurodegeneration partially by attenuating oxidative stress (Tsunemi et al., 2012). A recent study revealed that a variant of PGC-1α is associated with an earlier age onset of HD (Weydt et al., 2014). Furthermore, mice lacking PGC-1α show mitochondrial function impairment and HD features such as uncontrolled movement and striatal degeneration (Lin et al., 2004). Taken together, this evidence indicates that PGC-1α is a key factor in mediating neuronal death through oxidative stress induced by mHTT.

## mHTT-Induced Oxidative Stress and Potential Therapeutic Targets for HD

Multiple pathways that contribute to oxidative stress in HD have been described, including elevated NOX activity, oxidation of mitochondrial enzymes, the disturbance of active vitamin B6 and the activation of the antioxidant defense system (Table 1).

**Table 1:** Major published oxidative stress-associated processes and mediators of Huntington’s disease (HD) pathogenesis.

Processes and mediators	Model system	Type of mHTT	Description and consequence	References

mtDNA damage	Mouse; Striatal immortalized neuronal cell	Human HTT exon1: 115–150Q; 111Q	mHTT increases mitochondria-generated ROS and decreases mtDNA abundance.	Yang et al. (2008) and Siddiqui et al. (2012)
mtDNA depletion	Mouse and human HD patient	Human HTT exon1: 144Q; ~160Q	mHTT results in a lower copy number of mtDNA and increases the vulnerability of the striatum.	Petersen et al. (2014) and Hering et al. (2015)
Ca^2+^ imbalance	Rat cortical neuron	Truncated N-terminal HTT: 150Q	mHTT induces Ca^2+^ leakage through the Ca^2+^ channel RyR, further resulting in opening of the mPTP, which contributes to mitochondrial oxidative stress.	Suzuki et al. (2012)
p53	HEK293 cell; Mouse and human HD patient	Human HTT exon1^+/−^ proline-rich region: 103Q; N-terminal HTT: 86Q	p53 promotes expression of mHTT and mediates mHTT-induced mitochondrial dysfunction; the mHTT-p53 interaction suppresses transcription of p53-regulated genes.	Steffan et al. (2000) and Bae et al. (2005)
PGC-1α	Mouse and striatal cell culture	Human HTT exon1 and Mouse HTT: 111Q	mHTT reduces PGC-1α transcription and activity and suppresses downstream targets of PGC-1α, such as ROS defense factors.	Chaturvedi et al. (2012)
Drp1	Human HD patient; mouse; rat cortical neuron	Human mutant HTT; full-length human mHTT: 97Q; Human HTT exon1: 46 and 97Q	mHTT interacts with Drp1, disrupting the balance of the mitochondrial fission–fusion processes.	Shirendeb et al. (2011a); Shirendeb et al. (2011b) and Song et al. (2011)
NOX	Human HD patient and mouse	Mouse HTT: 140Q	Higher levels of brain NOX activity are observed in HD, whereas NOX inhibitors reduce the ROS level and neuronal death.	Valencia et al. (2012)
Mitochondrial enzymes	Human HD patient and mouse	Human HTT exon1: 94Q	Mitochondrial enzymes are oxidatively modified, with decreased catalytic activity and energy deficiency in individuals with HD.	Sorolla et al. (2010)
Vitamin B6	Human HD patient and mouse	Human HTT exon1: 94Q	Oxidation of pyridoxal kinase and antiquitin 1 decreased the availability of active vitamin B6, leading to the disturbances of neurotransmitters.	Sorolla et al. (2010)
Kynurenine pathway	Yeast	Human HTT exon1 lacking proline-rich region: 103Q	mHTT induces cytotoxicity through the kynurenine pathway and its intermediate product QUIN, which triggers striatal neuronal death via a cascade of events, such as oxidative stress.	Giorgini et al. (2005); Stoy et al. (2005) and Jamwal et al. (2015)
Prx1	PC12 cell	Human HTT exon1: 103Q	mHTT affects expression of the antioxidant protein Prx1, disturbing the ROS clearance system.	Pitts et al. (2012)
GPxs	Yeast	Human HTT exon1 lacking proline-rich region: 103Q	These antioxidant enzymes protect against ROS production and suppress mHTT toxicity.	Mason et al. (2013)
RQC	Yeast	Human HTT exon1 precence/absence of proline-rich region: 103Q	RQC system regulates mHTT compartmentalization and nucleocytoplasmic translocation that is associated with polyQ cytotoxicity.	Yang et al. (2016) and Zheng et al. (2017)

NOX, which produces ROS, has been used as an indicator for the ROS level *in vivo*. Researchers detected higher levels of brain NOX activity in post mortem HD cortex and striatum than in controls, particularly, NOX2, which localizes at plasma membrane lipid rafts, directly responsible for ROS level and survival in HD mice (Valencia et al., 2012). Conversely, NOX activity, ROS level and neuronal cell death were significantly reduced when cells were treated with NOX inhibitors (Valencia et al., 2012).

Oxidative modification of proteins is a main aspect of ROS-mediated cytotoxicity *in vivo*, which encouraged researchers to explore the unique oxidative proteins in HD individuals. Indeed, by comparing HD and control samples, Sorolla et al. (2010) identified 13 proteins that were oxidatively modified, including mitochondrial enzymes. Modification of these enzymes decreased their catalytic activity, consistent with the observation of energy deficiency in HD (Sorolla et al., 2010). More importantly, oxidation of pyridoxal kinase and antiquitin 1 decreased the availability of pyridoxal 5-phosphate, the active form of vitamin B6, leading to neurotransmitter disturbances, which is critical in HD pathology (Sorolla et al., 2010). Another study that focused on the proteomic levels of post mortem human brain samples obtained from striatum and cortex identified a significant increase in the level of antioxidant defense proteins, including peroxiredoxins (Prxs) 1, 2 and 6 and Gpx-1 and 6 in individuals with HD (Sorolla et al., 2008). Energy metabolism defects have been consistently proposed to contribute to the pathogenesis of HD (Jenkins et al., 1993; Brouillet et al., 1995; Gu et al., 1996). These findings suggest that oxidative stress and damage to specific macromolecules are increased in HD patients and that the antioxidant defense system alleviates oxidative stress and damage during the HD progression.

Thus far, the connection between oxidative stress and HD is not yet fully understood. It has been suggested that mHTT induces cytotoxicity through the kynurenine pathway (Giorgini et al., 2005; Stoy et al., 2005). In this respect, it has been shown that inhibition of the kynurenine pathway reduces the production of quinolinic acid (QUIN), ROS level, and cytotoxicity induced by mHTT (Giorgini et al., 2005). QUIN triggers striatal neuronal death via a cascade of events including oxidative stress, which is closely related to mitochondrial dysfunction. Recently, Jamwal et al. (2015) observed that spermidine, a molecule with free radical scavenging and anti-inflammatory properties, significantly attenuated the pathological alterations caused by QUIN treatment in rats (Jamwal et al., 2015). Similarly, sulforaphane has been proven to be effective in preventing mitochondrial dysfunction induced by QUIN in the rat striatum (Luis-García et al., 2016). Therefore, it is well worth examining the potential neuroprotective role of spermidine and sulforaphane in HD.

Furthermore, expression of mHTT triggered a decrease in the expression of the antioxidant protein Prx1. The same study also showed that treatment by dimercaptopropanol, a thiol-based antioxidant, rescued the cytotoxicity induced by mHTT and the expression level of Prx1 (Pitts et al., 2012). These results suggest that mHTT can exert its toxic effect by disturbing the ROS clearance system and document the potential of thiol-based antioxidants as drugs for HD treatment. However, some of the antioxidants do not seem to attenuate HD symptoms, which is likely because HD-induced ROS damage mainly occurs at the mitochondria. Indeed, inducing a synthetic mitochondria-specific antioxidant XJB-5-131, which targets mitochondria, remarkably suppressed oxidative mtDNA damage, restored the copy number of mtDNA, and alleviated the pathophysiology in HD animals (Xun et al., 2012; Polyzos et al., 2016). In addition, oxidation of cysteines within mHTT promoted the formation of soluble HTT oligomers, which are mediators of HD pathogenesis, and thereby interfered with the clearance of soluble mHTT, leading to enhanced mHTT cytotoxicity (Fox et al., 2011).

## Yeast Models Provide Insights Into Oxidative Stress in HD

Yeast lacks an HTT ortholog, and thus, its value for studying cellular communication is limited due to the absence of axonal and synaptic structures within a yeast bud (Khurana and Lindquist, 2010), as well as the lack of neural transmitters and the corresponding receptors (Khurana and Lindquist, 2010). As such, the suitability of the yeast model should be carefully taken into consideration before application to the study of HD. However, expression of mHTT fragment in yeast reproduces two major features of HD (i.e., protein aggregation and mHTT fragment-induced cytotoxicity). Moreover, many findings in yeast models were confirmed in higher model systems, such as mammalian cell lines, *C. elegans*, mice and even human patients. The insights gained from the studies in yeast have contributed greatly to our understanding of the pathogenesis of HD (Winderickx et al., 2008; Khurana and Lindquist, 2010; Mason and Giorgini, 2011; Fruhmann et al., 2017). As most mediators and pathways of energy metabolism are highly conserved in eukaryotes, it is not surprising to see that mHTT fragment induces oxidative stress in yeast. Here, we discuss the oxidative stress response of mHTT fragment and related findings in yeast models.

A toxic version of the mHTT fragment expression system lacking the proline-rich region in yeast established by the lab of Sherman and Lindquist (Krobitsch and Lindquist, 2000; Meriin et al., 2002; Willingham et al., 2003) revealed numerous molecular insights into mHTT fragment-induced toxicity, including findings regard to oxidative stress. In this yeast model, ROS accumulation was enhanced, and abnormal mitochondrial morphology and distribution in cells expressing mHTT fragment were observed. These findings revealed that the polyQ-mitochondria interaction interferes with mitochondrial function and increases ROS production (Solans et al., 2006). Screening the yeast deletion collection for suppressors of mHTT fragment-induced toxicity revealed that kynurenine 3-monooxygenase (Bna4) deficiency rescues cell viability (Giorgini et al., 2005). Metabolites of the kynurenine pathway have been shown to induce oxidative stress and neurological disorders (Pawlak et al., 2009; Parasram, 2018), which is associated with HD pathophysiology (Stoy et al., 2005). Another suppressor screening with gene overexpression, instead of loss-of-function, identified GPxs as potent suppressors of mHTT fragment toxicity (Mason et al., 2013). GPx is an antioxidant enzyme that protects against ROS, thus, enhancing the GPxs capacity can alleviate mHTT fragment toxicity (Table 1). Treatment with ebselen, which mimics the antioxidant effects of GPx, showed a strong protective effect on HD model organisms (Mason et al., 2013). These findings shed light on the clinical potential of ebselen and other antioxidants for HD therapy. Another study in yeast also demonstrated a role for Sir2 in protecting cells from ROS. Sir2 levels increased dramatically when yeast cells suffer from oxidative stress from mHTT fragment expression or the environment. However, enhancing Sir2 expression by a Sir2 activator decreased mHTT fragment aggregation and the mHTT fragment-induced stress. On the other hand, SIR2 knock-out cells showed significant oxidative stress even in the early exponential phase (Sorolla et al., 2011). Overexpression of Hap4, a protein that regulates many genes encoding mitochondrial proteins in yeast, prevented respiratory defects and ameliorated polyQ expansion-induced cytotoxicity (Ocampo et al., 2010). Together, these studies demonstrate that mHTT fragment induces oxidative stress in yeast and that preventing such oxidative stress can be a good strategy to handle mHTT fragment cytotoxicity.

Recently, a technique that combines automated yeast genetics and high-content microscopy has been applied to study mHTT fragment localization, protein-protein interaction and aggregate morphology in yeast. Taking advantage of this powerful screening approach, Yang et al identified that both the ribosome quality control (RQC) system and Hsf1 regulate the compartmentalization of mHTT fragment containing the proline-rich region (Yang et al., 2016). A follow-up study by Zheng et al. (2017) demonstrated that the RQC system indeed regulates the nucleocytoplasmic translocation of mHTT exon-1 lacking the proline-rich region and that a defective RQC system leads to enhanced cytotoxicity of mHTT fragment. Interestingly, it has been shown that oxidative stress can cause damage to rRNAs inside the ribosome, and oxidized rRNA inhibits protein synthesis in the ribosome (Willi et al., 2018). In this respect, mHTT-induced oxidative stress might be a driving force for the impairment of RQC system.

## Concluding Remarks

More than two decades have passed since the first identification of the *HTT* gene, and mutation of this gene is responsible for HD pathogenesis. The mechanisms responsible for the development of the single gene mutation that induce this neurodegenerative disease are not yet fully understood, and to date, no effective therapeutic target has been developed. The major findings point towards a close relationship between HD pathogenesis and oxidative stress.

Although multiple factors are involved in regulating mHTT-mediated oxidative stress, there are indications that mitochondria play a key role. Both mtDNA damage and mtDNA depletion have been detected upon expression of mHTT (Yang et al., 2008; Napoli et al., 2013). Given the core role of mitochondria in respiration and energy production, mtDNA damage or depletion leads to increased levels of oxidative stress. Moreover, a defect in mitochondrial calcium handling was observed in HD patients (Panov et al., 2002). Failure of keeping a mitochondrial calcium balance triggers the opening of the mPTP and the subsequent events lead to the final stage of cell death. The connection between oxidative stress and HD is also supported by the interaction of mHTT with p53 or PGC-1α, which modulates the activity of mitochondria. NOX activity, oxidation of mitochondrial enzymes, the disturbance of active vitamin B6 and the activation of antioxidant defense system are also implicated in sensing oxidative stress from mHTT.

Increased oxidative stress is detected during HD pathogenesis, and the published literatures reviewed here favors the idea that expression of mHTT is the primary cause of HD and that mHTT induces cellular dysfunction via multiple pathways, including oxidative stress. In this aspect, oxidative stress is likely a downstream consequence of HD pathogenesis. Moreover, other neurodegenerative disorders such as Alzheimer’s disease and Parkinson’s disease are controlled by multiple genes that are associated with oxidative stress (Gandhi and Abramov, 2012; Kim et al., 2015).

Despite the intensive studies on HD pathogenesis and evidence showing that oxidative stress occurs during this process, the major pathways and the sequence of oxidative stress-associated events in HD pathogenesis are still not known. Future studies that strengthen the understanding of oxidative stress in HD pathogenesis will provide insights into drug development and identification of therapeutic targets, for instance, clarifying the connections among the known pathways involved in mHTT-induced oxidative stress, identifying oxidative stress associated key factors in HD pathogenesis, and determining the major aspects as well as the order of pathologic events that contribute to HD development. Overall, this review article provides an overview of the current studies linking oxidative stress and HD development.

## Author Contributions

BL, VF and JW conceived and outlined the article and JZ wrote the manuscript with the help from VF, JW and BL.

## Conflict of Interest Statement

The authors declare that the research was conducted in the absence of any commercial or financial relationships that could be construed as a potential conflict of interest.
